# A Post-Pandemic Surge: Sustained High Prevalence and Epidemiological Shift in Pediatric Epstein–Barr Virus Infection

**DOI:** 10.3390/pathogens15040361

**Published:** 2026-03-29

**Authors:** Huamei Li, Ran Tao, Wei Li, Shiqiang Shang

**Affiliations:** Department of Clinical Laboratory, Children’s Hospital, Zhejiang University School of Medicine, National Clinical Research Center for Children and Adolescents’ Health and Diseases, 3333 Binsheng Road, Hangzhou 310052, China

**Keywords:** children, COVID-19, Epstein–Barr virus, infection

## Abstract

The coronavirus disease 2019 (COVID-19) pandemic altered the infection patterns of various pathogens; however, its impact on pediatric active Epstein–Barr virus (EBV) infection has not been sufficiently investigated in large-scale studies, and its long-term effects remain unexplored. We analyzed 57,403 pediatric patients from January 2018 to December 2024. Positivity rates of active EBV infection remained consistently elevated throughout the two-year period following the onset of the COVID-19 pandemic, significantly exceeding both pre-pandemic and during-pandemic levels (5329 [22.83%] vs. 1866 [13.55%] vs. 2188 [14.40%], *p* < 0.001), with concomitantly higher viral loads (median: 8.00 × 10^3^ vs. 1.78 × 10^3^ vs. 1.88 × 10^3^ copies/mL, *p* < 0.001). Post-pandemic, patients with EBV more frequently presented with pneumonia and allergic dermatitis, and the 6–11-year-old group accounted for a higher proportion of cases. This study reveals a prolonged surge of pediatric active EBV infection after the pandemic, characterized by sustained high prevalence, an age shift toward school children, and evolving clinical features. In the post-pandemic era, heightened and sustained attention should be paid to EBV infection in children, particularly among school-aged children. The sustained high prevalence of pediatric active EBV infection in the post-pandemic period warrants further investigation into its underlying causes.

## 1. Introduction

The Epstein–Barr virus (EBV), also known as human herpesvirus 4, belongs to the gamma herpesvirus family and infects more than 90% of adults globally [1,2,3,4]. Primary infections usually occur during childhood or adolescence, and are correlated with socioeconomic status and environmental hygiene, with a trend toward later onset in developed countries [4]. Primary EBV infection in infants and young children is often asymptomatic or with atypical symptoms, whereas infection in adolescents and adults commonly develops into infectious mononucleosis (IM), characterized by fever, pharyngitis, and lymphadenopathy. EBV can also cause nasopharyngeal disease, lymphoma, systemic lupus erythematosus, multiple sclerosis, and rheumatoid arthritis [5,6,7,8,9,10]. After primary infection, EBV establishes latency in B lymphocytes and oropharyngeal epithelial cells. When host immunity is low, virus reactivation can occur, resulting in disease recurrence [11,12,13]. Both primary and reactivated infections are considered active infections. During active EBV infection, lytic replication in infected tissues (e.g., oropharyngeal epithelium and B cells) releases viral particles and their cell-free EBV DNA into the bloodstream. When combined with compatible clinical manifestations, the detection of EBV DNA in peripheral blood serves as a clinically valuable biomarker for identifying active EBV infection [14]. EBV is mainly transmitted through the saliva and is easily spread through close contact [15].

Since the onset of the coronavirus disease 2019 (COVID-19) pandemic in late 2019, the Chinese government has implemented non-pharmaceutical interventions (NPIs) such as social distancing, mask wearing, and frequent hand washing, which have reduced the spread of COVID-19 and other respiratory pathogens [16,17,18,19]. At the end of 2022, as the virulence of severe acute respiratory syndrome coronavirus 2 (SARS-CoV-2) declined, and these restrictions were lifted, leading to widespread SARS-CoV-2 infections that swept through virtually the entire Chinese population. From then until the end of 2024, there were approximately two small-scale outbreaks annually [20].

Against this backdrop of implemented NPIs and subsequent widespread SARS-CoV-2 infection, a unique opportunity arose to study their impact on pediatric EBV infection dynamics—its impact has not been sufficiently investigated in large-scale pediatric studies, and its long-term effects remain unexplored. In this study, real-time fluorescent quantitative polymerase chain reaction (PCR) was used to detect EBV-DNA in peripheral blood samples to analyze the characteristics of active EBV infection in the pre-COVID-19 (2018–2019), during-COVID-19 (2020–2022), and post-COVID-19 periods (2023–2024). The aim of this study was to clarify the epidemiological impact of NPIs and widespread SARS-CoV-2 infection on pediatric EBV, thereby providing a scientific basis for refining prevention and control strategies in the post-pandemic era.

## 2. Materials and Methods

### 2.1. Patient Selection

This retrospective study included pediatric patients who underwent EBV-DNA testing at the Children’s Hospital, Zhejiang University School of Medicine, between January 2018 and December 2024. The hospital is in Hangzhou, the economically vibrant capital of Zhejiang Province in eastern China. Data on patient demographics, clinical diagnoses, and laboratory findings were extracted from electronic medical records. The inclusion criteria were (1) age < 18 years and (2) clinically suspected EBV infection. The exclusion criteria were: (1) incomplete clinical data; (2) presence of severe underlying diseases; and (3) diagnosis of malignant tumors. For each patient, the first positive test result (if any) was included; otherwise, the first negative result was used. Patients were divided into five age groups: infants (<1 year); toddlers (1–2 years); preschool (3–5 years); school-age (6–11 years); and adolescents (≥12 years). This study was approved by the Ethics Committee of the Children’s Hospital, Zhejiang University School of Medicine (2024-IRB-0129-P-01), on 21 May 2024. Since the study utilized anonymized data obtained from previous clinical records, the requirement for informed consent was waived by the ethics committee.

### 2.2. Specimen Collection and Detection

Peripheral blood EBV-DNA was detected using real-time fluorescent quantitative PCR. Venous blood (2 mL) was collected into sterile EDTA-containing tubes and gently mixed. Plasma was separated by centrifugation, and total nucleic acids were extracted from 400 µL of plasma using the Nucleic Acid Extraction Kit (Shengxiang Biotechnology, Changsha, China) on an Automated Nucleic Acid Extraction System (Shengxiang Biotechnology, China) according to the manufacturer’s instructions. The extracted nucleic acids were eluted in 50 µL of elution buffer.

PCR was performed using the EBV-DNA Quantitative PCR Kit (Shengxiang Biotechnology, China) on an ABI 7500 instrument (Applied Biosystems, Waltham, MA, USA). Each 50 uL reaction mixture contained: 10 µL of extracted nucleic acid template, 2 uL of enzyme mix, 37 uL of PCR solution (containing primers, probes, dNTPs, Mg^2+^, and PCR buffer), and 1 uL of internal control, following the kit’s protocol. Each assay run included a negative control, a positive control, and a series of four quantitative standards (4 × 10^4^, 4 × 10^5^, 4 × 10^6^, and 4 × 10^7^ copies/mL) to generate a standard curve. The thermal cycling conditions were as follows: 50 °C for 2 min, 94 °C for 5 min, followed by 40 cycles of 94 °C for 15 s and 57 °C for 30 s. Viral loads (copies/mL) were calculated by comparing the cycle threshold values of the samples against the standard curve using the instrument’s software. According to the kit’s specifications, samples with EBV-DNA ≥ 400 copies/mL were defined as positive, while those <400 copies/mL were defined as negative.

### 2.3. Statistical Analysis

Statistical analyses were performed using SPSS version 19.0. Categorical variables were compared using the chi-square test or Fisher’s exact test. Differences in viral load across the three time periods were compared using the Kruskal–Wallis test. Multivariable logistic regression was used to identify factors associated with active EBV infection, with results expressed as adjusted odds ratios (ORs) and their 95% confidence intervals (CIs). Statistical significance was set at *p* < 0.05.

## 3. Results

### 3.1. Demographic Characteristics

A total of 57,403 pediatric patients aged 3 days to 18 years were included in the study (32,402 males and 25,001 females). Patients were grouped as follows: pre-COVID-19 period, n = 13,772 (January 2018 to December 2019); during-COVID-19 period, n = 20,263 (January 2020 to December 2022); and post-COVID-19 period, n = 23,368 (January 2023 to December 2024) (Table 1). Since EBV-DNA testing was not performed on patients from February to March 2020, only data from 2021 to 2022 (n = 15,198) were used for the during-COVID-19 period in the comparison.

The median (interquartile range) age of all study patients in the post-COVID-19 period (59 [29–96] months) was significantly higher than that in the pre- (40 [18–72] months) and during-COVID-19 (46 [23–72] months) periods (Table 1).

The median (interquartile range) age of EBV-DNA positive patients in the post-COVID-19 period (61 [39–84] months) was significantly higher than that in the pre- (44 [27–65] months) and during-COVID-19 (52 [34–72] months) periods (Table 2).

### 3.2. Variations in EBV-DNA Positivity Rates and Viral Loads

The number of positive EBV-DNA cases and the positivity rates differed significantly across the three periods (*p* < 0.001) (Table 1). Specifically, the positivity rate increased slightly from 13.55% (1866/13,772) in the pre-COVID-19 period to 14.40% (2188/15,198) during COVID-19 and then increased markedly to 22.83% (5329/23,368) in the post-COVID-19 period.

The median (interquartile range) EBV loads among the three periods were significantly different (*p* < 0.001) (Table 2). The median EBV load increased slightly from 1.78 × 10^3^ in the pre-COVID-19 period to 1.88 × 10^3^ during COVID-19 and then increased markedly to 8.0 × 10^3^ in the post- COVID-19 period.

### 3.3. Monthly Variation in EBV Infection

**Figure 1:**
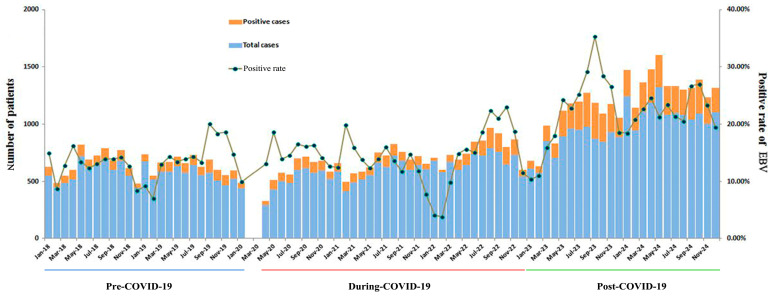
Monthly distribution of pediatric active Epstein–Barr virus infection in the pre-, during-, and post-COVID-19 periods (2018–2024). COVID-19, coronavirus disease 2019; EBV, Epstein–Barr virus.

As shown in Figure 1, the number of EBV-DNA tests and the number of positive cases were relatively similar between 2018 and 2021, with a slight increase in 2022 and a significant increase in 2023 and 2024. Positivity rates remained relatively stable from 2018 to 2022 but increased significantly in 2023 and 2024. With the exception of 2020 and 2021, positivity rates decreased in January or February with a minor peak in September or October each year. Small peaks occurred in May 2020 and February 2021.

### 3.4. Age Group Variations in EBV Infection

**Figure 2:**
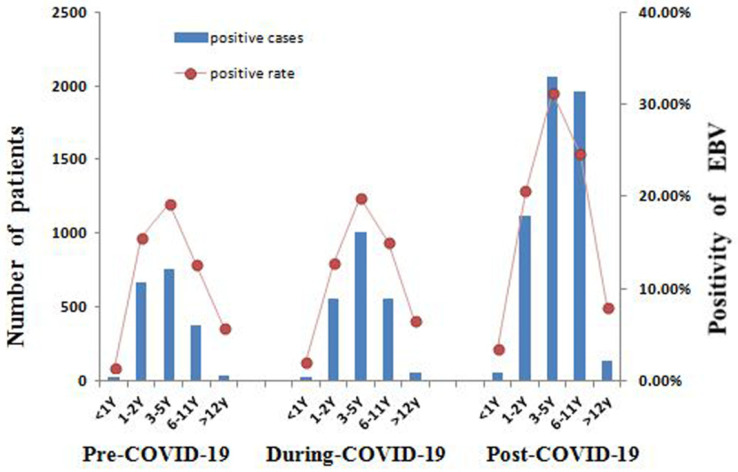
Age group distribution of pediatric active Epstein–Barr virus infection in the pre-, during-, and post-COVID-19 periods. COVID-19, coronavirus disease 2019; EBV, Epstein–Barr virus.

**Figure 3:**
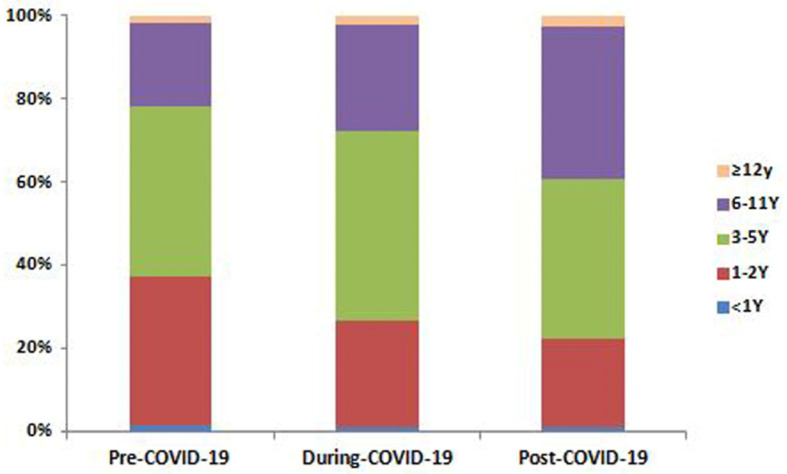
Proportion of pediatric active Epstein–Barr virus infection cases by age group in the pre-, during-, and post-COVID-19 periods. COVID-19, coronavirus disease 2019.

In the pre-COVID-19 period, EBV infection was the highest among the 1–2- and 3–5-year-old groups (positivity rate: 15.52% and 19.22%, respectively) (Figure 2). During COVID-19, this preschool predominance persisted. In the post-COVID-19 period, the case numbers and positivity rate in almost all age groups increased. The 3–5-year-old group maintained the highest absolute positivity rate (31.31%). Notably, the positivity rate in the 6–11-year-old group nearly doubled (from 12.64% to 24.59%). Furthermore, the proportion of EBV-positive patients in the 1–2-year-old group showed a marked decrease in the post-COVID-19 period, while that of the 6–11-year-old group increased significantly (Figure 3).

### 3.5. Factors Associated with EBV Infection

After adjusting for potential confounders, the multivariable logistic regression analysis confirmed the pandemic period, sex, and age group as independent factors associated with EBV infection (Table 3). Compared to the pre-COVID-19 baseline, the risk of infection was significantly higher in the post-COVID-19 period (OR = 1.81; 95% CI: 1.70–1.92; *p* < 0.001), but not during the pandemic itself (OR = 1.00; 95% CI: 0.94–1.07; *p* = 0.944). Furthermore, females had a slightly higher risk than males (OR = 1.10; CI: 1.05–1.15; *p* < 0.001). Using infants < 1 year as a reference, infection risk was sharply elevated in all older children (all *p* < 0.001), with the highest odds in the 3–5-year-old group (OR = 13.67; CI: 11.29–16.55). The risk was also notably higher in the 6–11-year-old (OR = 9.54; CI: 7.87–11.56) and 1–2-year-old groups (OR = 8.46, CI: 6.98–10.26), and the lowest was found in those aged ≥12 years (OR = 2.94, CI: 2.33–3.71).

### 3.6. Initial Diagnoses of EBV-DNA-Positive Pediatric Patients

Initial patient symptoms and disease types greatly varied. In addition to the classic IM triad (tonsillitis, lymphadenitis, and fever), many patients presented with only one component of the triad or other atypical symptoms, such as allergic dermatitis, pneumonia, upper respiratory tract infection, gastroenteritis, liver impairment, epilepsy, encephalitis, cytopenia, hemophagocytic syndrome, urinary system diseases, and cardiac injury.

After the pandemic, the proportions of patients initially diagnosed with pneumonia and allergic dermatitis were significantly higher than before and during the pandemic, while cytopenia decreased significantly (Table 4). The proportions of patients with IM and gastroenteritis during and after the pandemic were significantly higher than those before the pandemic; conversely, the proportions of patients with fever, tonsillitis, lymphadenopathy, and hemophagocytic syndrome decreased significantly.

## 4. Discussion

The COVID-19 pandemic has profoundly reshaped the epidemiology of respiratory infections. Our study identified a significant and prolonged surge in pediatric active EBV infections in the post-pandemic era. This surge was characterized not only by elevated incidence, positivity rates, and viral loads, but also by a distinct shift in age distribution toward school-aged children and an altered clinical spectrum—including a higher proportion of IM and complications such as pneumonia. These findings contrast with a previous report by Lan et al., which noted no significant changes [21], a discrepancy that may be attributed to regional variations and differences in sample size.

The post-pandemic surge in pediatric EBV infection is likely multifactorial. The NPIs led to an accumulation of EBV-naïve children, followed by a sharp rebound in social contact after the relaxation of these measures, which triggered a concentrated outbreak of primary infections—a plausible initial driver. However, a pivotal epidemiological observation in our study—the sustained high prevalence over the two years—suggests mechanisms beyond a simple rebound in primary infections. This pattern—though not proof—is most consistent with the hypothesis that SARS-CoV-2 infection may have reactivated latent EBV. The literature on adults supports SARS-CoV-2 as a trigger for EBV [22,23,24]. In our pediatric cohort, recurrent, small-scale waves of SARS-CoV-2 infection following the initial major wave could have repeatedly reactivated latent EBV, thereby sustaining an elevated baseline of active EBV infection throughout the subsequent observation period. The underlying mechanism by which COVID-19 triggers EBV reactivation may be related to immune dysregulation after infection [25,26,27,28]. It must be acknowledged that, while supported by our epidemiological data and the existing literature, the reactivation hypothesis remains speculative and lacks direct causal evidence in children. Future prospective studies that concurrently track SARS-CoV-2 infection history and perform testing for both EBV serology and viral load are needed to establish causality. Confirming this link would not only validate our hypothesis but also underscore the clinical relevance of a COVID-19 history in the management of severe or recurrent pediatric EBV illness.

Our study revealed a significant increase in EBV infections among children aged 6–11 years after the COVID-19 pandemic. The infection pattern shifted from a pre-pandemic predominance in 1–2- and 3–5-year-olds to a post-pandemic predominance in 3–5- and 6–11-year-olds. This shift may be explained by pandemic control measures, which prevented many young children from being infected with EBV at an early age as in previous years, thereby delaying the age of primary infection in the susceptible population. Additionally, while EBV infection in young children is often asymptomatic, older children are more likely to develop symptomatic diseases such as IM, leading them to seek medical attention. Therefore, in the post-COVID-19 pandemic era, in addition to focusing on infections in young children, attention should be paid to the risk of EBV infection in school-aged children.

We observed that the EBV infection rate peaked around September and declined around January, consistent with the findings of Liu et al. [29]. The September peak may be attributed to the summer vacation in China, during which children spend more time with their families and participate in social gatherings that increase viral exposure. The decline in January may be due to reduced social activities during winter, potential competition, or immune interference from other seasonal viral infections. The absence of these clear trends in 2020 and 2021, along with minor peaks in May 2020 and February 2021, may be linked to COVID-19 restrictions, remote learning, and prolonged household contact during lockdowns. Changes in clinical presentation were also observed: compared to the pre-pandemic and during-pandemic periods, the proportion of patients diagnosed with pneumonia and allergic dermatitis significantly increased after the pandemic, which may be related to immune dysregulation and respiratory system impairment following COVID-19.

Notably, unlike common respiratory viruses (e.g., influenza, RSV, and adenovirus), EBV positivity rates did not decline during the period of strict NPIs, a finding consistent with other pediatric studies [30]. This divergence underscores the distinct transmission ecology of EBV: primarily through saliva and close contact rather than via droplets and aerosols. Consequently, NPIs such as masking and distancing had limited impact on EBV spread; indeed, prolonged household contact during lockdowns may have even facilitated within-family transmission through behaviors such as kissing, sharing utensils, or caregiver pre-chewing of food [31,32,33].

Our study has several limitations. The single-center, retrospective design may introduce selection bias and limit generalizability. Furthermore, blood EBV-DNA detection indicates active infection but cannot distinguish primary infection from reactivation, and serological testing was not routinely available in this retrospective setting. Furthermore, data on the history and timing of SARS-CoV-2 infections in the study population were not available, which restricts our ability to directly test the proposed reactivation hypothesis.

## 5. Conclusions

Our study demonstrates a significant and prolonged post-pandemic surge of active EBV infection among Chinese children, characterized by higher viral loads, a distinct age distribution shift toward school-aged children, and an increased proportion of pneumonia and allergic dermatitis as initial diagnoses. In the post-pandemic era, heightened and sustained attention should be paid to EBV infection in children, particularly among school-aged children. The sustained high prevalence of pediatric active EBV infection in the post-pandemic period warrants further investigation into its underlying causes.

## Figures and Tables

**Table 1:** Total patient characteristics and the positivity rates of Epstein–Barr virus DNA in pre-, during-, and post-COVID-19 periods.

Characteristic	Pre-COVID-19 (2018–2019) (n = 13,772)	During COVID-19 (2021–2022) (n = 15,198)	Post-COVID-19 (2023–2024) (n = 23,368)	*p*-Value
Male sex, n (%)	7953 (57.75)	8588 (56.51)	13,215 (56.55)	>0.05
Age, median (IQR), month	40 (18, 72)	46 (23, 72)	59 (29, 96)	<0.001
Age groups, n (%)				<0.001
<1 year, n (%)	1967 (14.28)	1320 (8.69)	1644 (7.04)	
1–2 years, n (%)	4299 (31.22)	4359 (28.68)	5457 (23.35)	
3–5 years, n (%)	3959 (28.75)	5054 (33.25)	6576 (28.14)	
6–11 years, n (%)	3007 (21.83)	3690 (24.28)	7960 (34.06)	
≥12 years, n (%)	540 (3.92)	775 (5.10)	1731 (7.41)	
EBV DNA positivity, n (%)	1866 (13.55)	2188 (14.40)	5329 (22.83)	<0.001

IQR, interquartile range; EBV, Epstein–Barr virus.

**Table 2:** Characteristics and DNA loads of the Epstein–Barr virus DNA-positive pediatric patients in the pre-, during-, and post-COVID-19 periods.

Characteristic	Pre-COVID-19 (2018–2019) (n = 1866)	During COVID-19 (2021–2022) (n = 2188)	Post-COVID-19 (2023–2024) (n = 5329)	*p*-Value
Male sex, n (%)	1039 (55.68)	1165 (53.24)	2911 (54.62)	>0.05
Age, median (IQR), month	44 (27, 65)	52 (34, 72)	61 (39, 84)	<0.001
EBV DNA loads				<0.001
Median (copies/mL)	1.78 × 10^3^	1.88 × 10^3^	8.0 × 10^3^	
IQR (copies/mL)	7.43 × 10^2^–5.06 × 10^3^	8.68 × 10^2^–5.15 × 10^3^	2.33 × 10^3^–2.31 × 10^4^	

IQR, interquartile range; EBV, Epstein–Barr virus.

**Table 3:** Multivariable logistic regression analysis of factors associated with EBV infection.

Characteristic	Adjusted OR ^†^	95% CI	*p*-Value
Period (Reference: Before COVID-19)			
During COVID-19	1.00	0.94–1.07	0.944
Post-COVID-19	1.81	1.70–1.92	<0.001
Sex (Reference: Male)			
Female	1.1	1.05–1.15	<0.001
Age Group (Reference: <1 year)			
1–2 years	8.46	6.98–10.26	<0.001
3–5 years	13.67	11.29–16.55	<0.001
6–11 years	9.54	7.87–11.56	<0.001
≥12 years	2.94	2.33–3.71	<0.001

EBV, Epstein–Barr virus; OR, odds ratio; CI, confidence interval. ^†^ Adjusted for period, sex, and age group.

**Table 4:** Variations in initial diagnoses of Epstein–Barr virus DNA-positive pediatric patients in the pre-, during-, and post-COVID-19 periods.

Initial Diagnoses	Pre-COVID-19 (2018–2019) (n = 1866)		During COVID-19 (2021–2022) (n = 2188)		Post-COVID-19 (2023–2024) (n = 5329)		
	n	Percentage %	n	Percentage %	n	Percentage %	*p* Value
Infectious mononucleosis	796	42.66	1251	57.18	2969	55.71	<0.001
Fever	347	18.60	210	9.60	532	9.98	<0.001
Pneumonia	117	6.27	115	5.26	473	8.878	<0.001
Tonsillitis	213	11.41	130	5.94	367	6.89	<0.001
Upper respiratory infection	113	6.06	104	4.75	285	5.35	<0.001
Lymphadenopathy	131	7.02	76	3.47	236	4.43	<0.001
Cytopenia	74	3.97	93	4.25	90	1.69	<0.001
Liver injury	27	1.45	24	1.10	65	1.22	0.21
Allergic dermatitis	20	1.08	25	1.14	113	2.12	<0.001
Gastroenteritis	7	0.38	21	0.96	48	0.90	0.003
Hemophagocytic syndrome	23	1.23	16	0.73	27	0.51	0.045
Cardiocerebrorenal diseases	19	1.34	21	1.51	102	1.9	0.003

## Data Availability

The original contributions presented in the study are included in the article; further inquiries can be directed to the corresponding author.
